# Preparation of Gel Forming Polymer-Based Sprays for First Aid Care of Skin Injuries

**DOI:** 10.3390/gels10050297

**Published:** 2024-04-25

**Authors:** Patrícia Alves, Diana Luzio, Kevin de Sá, Ilídio Correia, Paula Ferreira

**Affiliations:** 1 Chemical Engineering and Renewable Resources for Sustainability (CERES), Department of Chemical Engineering, University of Coimbra, 3030-790 Coimbra, Portugal; palves@eq.uc.pt (P.A.); diana_luzio@hotmail.com (D.L.); icorreia@fcsaude.ubi.pt (I.C.); 2 Faculty of Health Sciences, University of Beira Interior, Av. Infante D. Henrique, 6200-506 Covilha, Portugal; ringo.kesa@gmail.com; 3 Applied Research Institute, Polytechnic Institute of Coimbra, Rua da Misericórdia, Lagar dos Cortiços—S. Martinho do Bispo, 3045-093 Coimbra, Portugal; 4 Research Centre for Natural Resources, Environment and Society (CERNAS), Polytechnic Institute of Coimbra, 3045-601 Coimbra, Portugal

**Keywords:** wound care, polymeric film, hemocompatibility, cytocompatibility

## Abstract

Currently, there are several types of materials for the treatment of wounds, burns, and other topical injuries available on the market. The most used are gauzes and compresses due to their fluid absorption capacity; however, these materials adhere to the surface of the lesions, which can lead to further bleeding and tissue damage upon removal. In the present study, the development of a polymer-based gel that can be applied as a spray provides a new vision in injury protection, respecting the requirements of safety, ease, and quickness of both applicability and removal. The following polymeric sprays were developed to further obtain gels based on different polymers: hydroxypropyl cellulose (HPC), polyvinyl pyrrolidone (PVP) and hydroxypropyl methylcellulose (HPMC) using polyethylene glycol (PEG) as a plasticizer. The developed sprays revealed suitable properties for use in topical injuries. A protective film was obtained when sprayed on a surface through a casting mechanism. The obtained films adhered to the surface of biological tissue (pig muscle), turning into a gel when the exudate was absorbed, and proved to be washable with saline solution and contribute to the clotting process. Moreover, biocompatibility results showed that all materials were biocompatible, as cell viability was over 90% for all the materials.

## 1. Introduction

Every day, accidents cause unintentional topical injuries (e.g., abrasions, lacerations, cuts, bites, or burns) with variable degrees of severity. Therefore, a material that can easily provide wound protection is necessary in these cases. Depending on the type of injury, wound treatment may be performed at home or at the hospital. Currently, for small-scale domestic accidents, the use of wound dressings is a common procedure. However, in cases of increased severity, medical assistance by qualified professionals may be required. The usual procedure by qualified medical assistance personnel generally involves the application of saline solution for wound washing and posterior cleaning with gauze swabs. These materials are used as first aid until arriving at the hospital for appropriate treatment of the wounds.

Nowadays, research on new materials for wound treatment has allowed the evolution and conception of products with different characteristics and functionalities. The main goals of the existing products on the market are the protection of lesions on the skin’s surface and the ability to absorb wound exudates [1].

Amongst the most well-known types are polyurethane (PU) foams [2] which are semi-permeable, non-adherent, and hydrophilic. These dressings are recommended for wounds with moderate bleeding. These materials may be used in primary (direct contact with the wound) or secondary (used to cover a primary material for protection purposes) treatment, for example, alginate dressings [3]. PU foams function like a soft layer that protects the affected area, providing an environment prosperous to skin regeneration. However, PU foams present some drawbacks, such as their opacity, which limits wound visualization, and their lack of self-adhesiveness, therefore requiring other materials that allow their fixation [4].

Another class of wound dressings, hydrogels, are composed of hydrophilic polymers with high absorption capacity, which allows the interaction and exchange of fluids between the material and the wound surface [5]. Thus, they do not easily adhere to the wound surface, and because of the hydrogels’ high water content, it is possible to cool the wound, relieving the pain and discomfort of the patient while maintaining a suitable environment for healing, which helps in wound protection, promoting an efficient regeneration [6].

Another type of dressing is made from hydrocolloids [7]. Hydrocolloids contain gel forming agents (hydrophilic colloidal particles), generally gelatine or sodium carboxymethylcellulose, combined with an external layer, generally based on a PU. These dressings are, therefore, composed of two layers with distinct functions. The external layer protects the wound from environmental aggressions, such as bacteria, and the bottom layer is in direct contact with the wound and is responsible for the absorption of wound exudates. This absorbent layer of the dressing, when in contact with the wounds’ moisture, forms a smooth gel. This gel material contributes to the maintenance of moisture on the wound and protects the granulation tissue, enhancing wound healing [8].

Polymer-based sprays that are able to create a film barrier over a wound have already been developed [9]. These solutions allow simultaneous wound disinfection and protection, providing immediate action against external microorganisms with minimum discomfort for the patient. A commercially available spray known as Elastoplast^®^ (Beiersdorf AG, Hamburg, Germany) is composed of ethanol, water, dimethyl ether, and PU/acrylic copolymer [10]. When the polymer solution is applied to the skin surface, a flexible, water-resistant film is formed. This film can promote the protection of the wound against external conditions for a few days, which is essential for the beginning of the process of skin regeneration. However, this product is difficult to remove, and its fluid absorbance capacity is limited. The film dissolves after a few days, but to remove the film earlier, a solvent based on isopropyl alcohol or ethyl acetate is needed, damaging the wound and promoting new bleeding.

Nobecutan^®^ (INIBSA Laboratories, Sintra, Portugal) is another available option that allows simple and economical treatment. This spray is based on an acrylic resin and tetramethylthiuram disulfide (TMTD) (strong bactericidal and fungicidal) dissolved in ethyl acetate [11]. Nobecutan^®^ works in the same way as Elastoplast^®^, producing a transparent film with the capacity to protect the affected areas over several days. After healing and regeneration of the injuries, the film is spontaneously eliminated without causing additional damage [12].

Despite these advances, the developed products in the form of sprays have been focused on the medium-term treatment of injuries. In this work, the focus was the development of materials that allow a new approach to wound first-aid care. The goal was to obtain polymer-based sprays that are able to form films over the injury but that are easily removed by washing once healthcare personnel is available to perform definitive wound treatment. For this purpose, polymer selection was carried out according to predetermined requirements such as solubility in the selected solvent (ethanol), the ability to form films through the solvent evaporation method on the skin surface, and the capacity of the formed films to be soluble in aqueous solutions. Different formulations were developed based on the cellulose derivatives hydroxypropyl cellulose (HPC) and hydroxypropyl methylcellulose (HPMC) due to their ability to form films with high elasticity [13,14]. These materials are currently widely applied in several industrial fields, such as in the pharmaceutical industry for the encapsulation of tablets and capsules and in cosmetics, such as aerosols and hairspray [13]. Polyvinyl pyrrolidone (PVP) was also tested in formulation when combined with HPC [15,16]. PVP is widely used in several application areas, such as the pharmaceutical and food industries, due to its properties, such as solubility in water and polar organic solvents, as well as the ease of producing films with high elasticity [17,18]. In all cases, polyethylene glycol was added as a plasticizer in order to obtain films with increased flexibility [19]. The rheological behavior of the formulations was evaluated, and the solubility, thermal performance, hemocompatibility, and cytocompatibility of the obtained films were assessed.

## 2. Results and Discussion

### 2.1. Evaluation of Spraying Capacity and Adhesion to Biological Substrates

A qualitative assessment of characteristics such as the suitability of the formulations to be applied as sprays, their ability to originate homogeneous films, their detachability and flexibility, as well as their washability with saline solution was executed ex vivo over pig muscle tissue. Table 1 summarizes the overall conclusions concerning these features.

Overall, the prepared formulations were sprayed effortlessly over the surfaces and originated flexible films. Moreover, these films were not easily detached, showing a good adhesion to the surface. Concerning the adhesion to the biological tissue, it was verified that all samples were able to spread and adhere on the surface and that the resultant film was not easily removed. However, when washed with saline solution, all films turned into gels and, therefore, were efficiently removed, with the ones based on HPC-PVP being easier to displace.

### 2.2. Characterization Techniques

#### 2.2.1. Rheology

**Figure 1:**
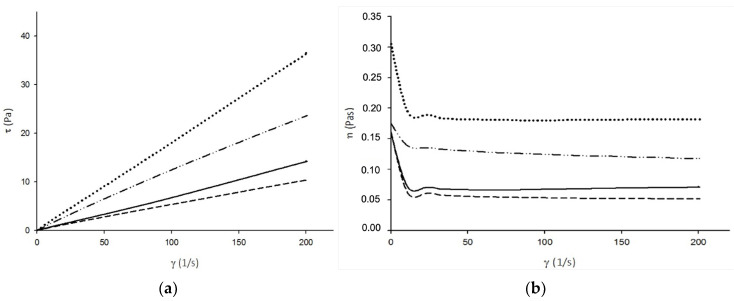
(**a**) Shear stress profiles according to the strain rate variation and (**b**) Viscosity profiles according to the strain rate variation for HPC-HPMC (3:3) (──); HPC-HPMC (4:4) (······); HPC-PVP (4:4) (-------); and HPC-PVP (5:3) (—··—).

The viscosity and the shear stress under the influence of the strain variation of the formulations were evaluated by rheology studies. Figure 1a,b show the obtained results. As illustrated in Figure 1a, the fluids exhibit a Newtonian behavior since the shear stress is directly proportional to the deformation rate, which is the basic characteristic of this type of fluid [20]. Referring to Figure 1b, viscosity curves indicate this same Newtonian fluid behavior since viscosity is not affected by the variations in the deformation rate and remains constant. These results indicate that all formulations have an ideal behavior that is independent of the deformation rate applied [20].

Comparing the viscosity results of the HPC-HPMC (4:4) and HPC-PVP (4:4) formulations, HPC-HPMC (4:4) showed a higher viscosity than HPC-PVP (4:4) due to the different viscoelastic properties of HPMC and PVP, which directly affect the viscosity of the final formulations. HPC is known to present a low viscosity [21], while PVP has the tendency to increase fluid viscosity while not changing the surface tension [22]. Additionally, some authors reported that by increasing the HPMC concentration, a rise in the viscosity could be observed [21]. Thus, the rheological behavior of the formulations was influenced by the polymers used and their concentrations. Considering that according to the literature [23], for proper spraying of polymer solutions, the formulations’ viscosity should present values between 0.014 Pas and 0.2 Pas, it can be concluded that all formulations prepared exhibited viscosities suitable for the intended purpose.

#### 2.2.2. Solubility Test in Saline Solution

**Figure 2:**
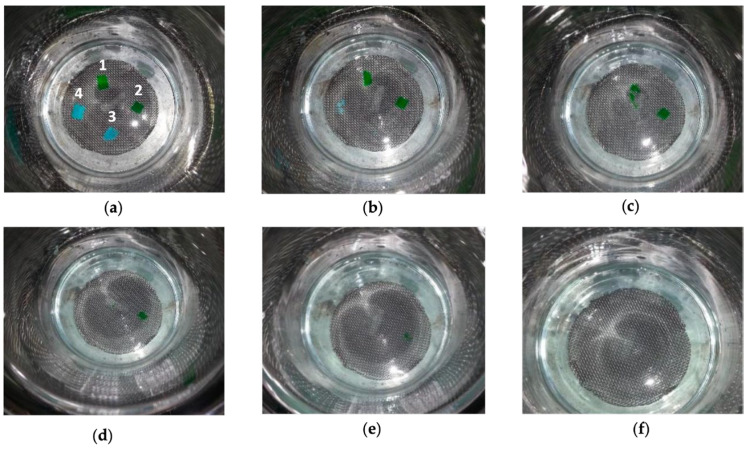
Solubilization of the films: 1—HPC-HPMC (3:3); 2—HPC-HPMC (4:4); 3—HPC-PVP (4:4); and 4—HPC-PVP (5:3) in physiological saline over time. (**a**) t = 0 min; (**b**) t = 4 min; (**c**) t = 8 min; (**d**) t = 12 min; (**e**) t = 16 min; and (**f**) t = 20 min.

The solubilization test was carried out at room temperature for 20 min and the evolution of the solubilization process was observed every 4 min. Figure 2 shows the sequence of events during the evaluation of the solubility of the films in a physiological saline solution.

The results showed that the films exhibited different solubilities according to the differences in the films’ formulations. Films containing PVP in their formulation showed a very high solubility capacity in saline solution, with dissolution occurring within the first 4 min for the formulation with the higher proportion of PVP (HPC-PVP (4:4)). Decreasing the amount of PVP in the formulation led to an increase in the solubilization time due higher solubility of PVP in aqueous medium [24].

When compared to PVP-based films, HPMC formulations showed higher solubility times due to this polymer’s lower solubility in aqueous solutions than PVP since HPMC slowly dissolves in cold water to form a viscous solution [25]. Therefore, during the test, HPMC-based films initially formed a gel, remaining in place for about 10 to 20 min before total solubilization. Thus, HPC-PVP (4:4) and HPC-PVP (5:3) films can be easily removed faster from affected areas with saline solution (in 4 to 6 min). Table 2 shows the solubilization times for each film.

However, the high solubilization capacity can become a limitation when applied over wounds with increased bleeding or exudate production. Therefore, due to their gel-forming capacity and lower solubility, HPC-HPMC (3:3) and HPC-HPMC (4:4) films will be able to stay in place longer as they will not immediately solubilize, which may be beneficial for these kind of topical lesions.

#### 2.2.3. Dynamic–Mechanical Thermal Analysis

**Figure 3:**
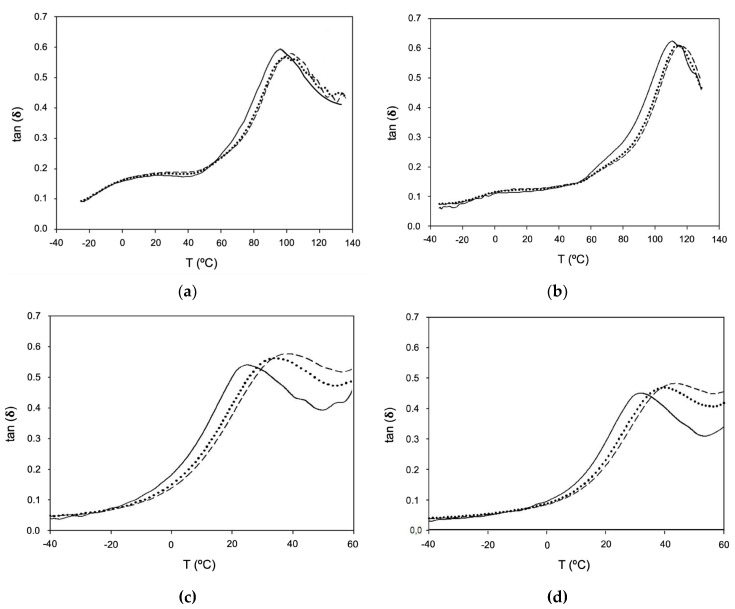
Traces of tan δ as a function of temperature for: (**a**) HPC-HPMC (3:3); (**b**) HPC-HPMC (4:4); (**c**) HPC-PVP (4:4); and (**d**) HPC-PVP (5:3). Frequencies: 1 Hz (──); 5 Hz (·······); and 10 Hz (——).

The thermal and viscoelastic properties of the films were analyzed by dynamic–mechanical thermal analysis (DMTA). DMTA results obtained for the films are shown in Figure 3a–d. HPMC-based films had significantly higher T_g_ values of 95 and 111 °C (Figure 3a,b), respectively, when compared to PVP-based films with T_g_ values of 22 and 32 °C (Figure 3c,d, respectively). The literature reports that T_g_ is directly affected by the chemical structures of the repeating units of the polymers, such as polar interactions, crosslinking between chains, and rigid groups (aromatic groups, ether hydrocarbons, etc.) in the main chains, among others [26]. Thus, it can be concluded that HPMC and PVP polymers combined with HPC allow to obtain films with very distinct thermal properties. HPC has amorphous zones with low T_g_ in parallel with crystalline domains, resulting in a structure with high mobility and, consequently, low T_g_ value [21]. HPMC has aromatic and ether groups in the backbone structure, limiting the movement of chains, and consequently, the films have high rigidity, which justifies the higher T_g_ values [27]. Another factor contributing to higher T_g_ is the possibility of hydrogen bonds between HPC and HPMC polymers, reducing the mobility of the chains and increasing stiffness. In contrast, some authors found PVP T_g_ to be between 54~175 °C under different water content levels [28,29], along with the fact that PVP polymer lacks rigid groups in the backbone chain, allowing higher flexibility [30]. Therefore, as expected, Figure 3c,d shows that with the increase in frequencies, an increase in T_g_ is observed due to the relaxation of the polymer chains.

#### 2.2.4. Surface Free Energy Assessment

The purpose of this work was to develop a film-forming material to be applied over skin and bleeding surfaces for the temporary treatment of wounds. For this reason, spreading and adhesion between those materials and the referred surfaces need to be assessed. According to the thermodynamic principle of adhesion, in order to achieve adhesion, the surface energy of the adhesive must be equal or inferior to that of the substrate [31]. Therefore, the surface tension of the liquid formulations, as well as the surface energies of the films obtained from each of them, were measured in order to compare them with the ones of blood and skin, respectively.

Table 3 summarizes the obtained results for the surface tensions and surface energies ($\gamma_{s}$) as well as their polar ($\gamma_{s}^{p}$) and dispersive ($\gamma_{s}^{d}$) components for each sample. The reported values of surface energy for dry skin [31] and surface tension for blood [32] are also presented.

As can be observed in Table 3, the surface tensions of all liquid formulations were lower than those of blood. Considering these results, it is expected that the adhesion forces between the liquid formulations and a bleeding surface will overcome the cohesive forces in any of the polymeric solutions. Therefore, spreading of the polymeric formulation and consequent wetting of that surface is prone to occur. Moreover, surface energies of the films resultant from any of the formulations are also inferior to the ones of dry skin while remaining lower than the surface tension of blood. Also, considering that the polar components of films’ surface energies are noteworthily higher than their dispersive components, the main interactions between the films and substrates will occur between permanent dipoles, induced dipoles, and dipoles and hydrogen bonds. However, skin, being composed of lipidic components, is primarily hydrophobic, and its dispersive component is the predominant one [33]. This fact could compromise adhesion due to the polar nature of the samples since a more similar ratio of the dispersion and polar components leads to higher interactions between the phases and, therefore, better adhesion. Nevertheless, the films will be prepared over moist surfaces, meaning that the polar component will be significantly higher due to the presence of water. Consequently, adhesion will be more efficient due to the increased intermolecular interaction between the films and the moist surface.

#### 2.2.5. Characterization of the Biological Properties

##### Thrombogenicity Assessment

The thrombogenicity evaluation allows assessing whether a blood thrombus is formed when blood directly contacts the materials’ surfaces. Thrombus formation is a complex process that initiates with platelet adhesion and activation and ends with the activation of the coagulation cascade thrombin generation and fibrin polymerization [34]. This process begins within 30 s of blood/material contact and generates a stable thrombus within 3–5 min. During this work, this assessment was performed by gravimetry after 4 min of blood/surface contact. This period of incubation was established considering solubilization times previously obtained for the samples in the solubility test in saline solution (Section 2.2.2).

**Figure 4:**
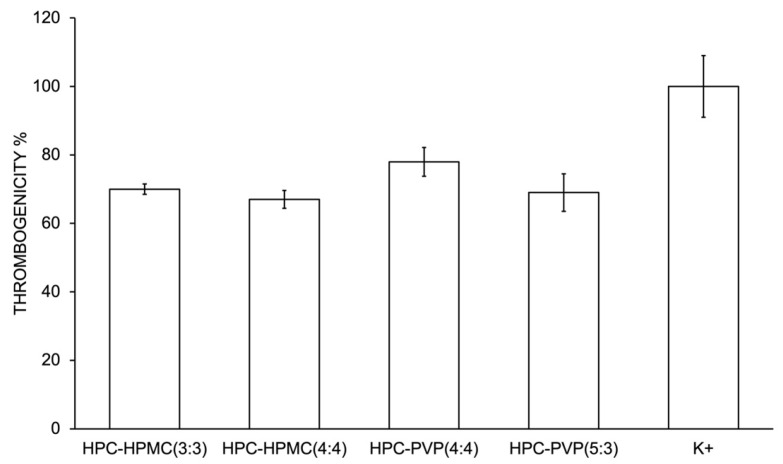
Mass values (%) of the thrombus formed on the surface of the membranes after 4 min of blood contact. Values obtained represent the average of three samples.

Analyzing the results presented in Figure 4, it is possible to confirm that the films exhibit a thrombogenic character, presenting mean values of thrombogenicity between 67% and 78% when compared with the positive control (glass).

These results are in line with those obtained for the surface energy values. According to the literature, the thrombogenicity of the material is directly related to its surface energy [35]. In fact, previous studies reported that materials that present low surface energies tend to be more thrombogenic since protein adhesion to those surfaces occurs in higher quantities, promoting the coagulation process [36,37,38]. Considering this work, the hemostatic character attributed to the thrombogenicity of these materials would largely contribute to decreased bleeding, promote coagulation, and, consequently, assist in the temporary treatment of the wound until further intervention.

##### Evaluation of Materials Cytotoxicity

**Figure 5:**
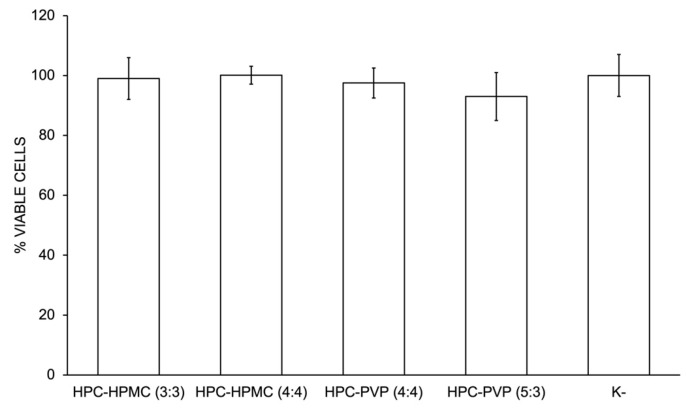
Evaluation of the cellular activity after 1 day of incubation with each sample and of the negative (K^−^) control. Each result is the mean ± standard error of the mean of five independent experiments.

For the materials’ cytocompatibility evaluation, human fibroblasts were seeded in direct contact with the polymeric films, and their viability after 1 day of incubation was evaluated using the MTS assay. This period of contact was chosen based on the temporary profile of use intended for the prepared materials. The results obtained are shown in Figure 5.

Results showed that after 1 day of incubation, despite solubilizing, all materials are biocompatible, with cellular viabilities above 90%. These results demonstrated that none of the formulations had a critical effect on cell integrity or viability, which is a critical feature for their application in wound protection.

## 3. Conclusions

This work was developed in order to obtain polymer-based materials able to perform as a temporary and easily removable barrier for wound protection. Different formulations based on HPC, HPMC, and PVP were prepared, and their properties were evaluated. All the formulations formed films when sprayed over a surface. Once formed, those films adhered to the tested biological tissue (pig muscle) and were easily washed under irrigation with saline solution and using a gauze swab due to their solubility in water. The rheologic evaluation showed that the materials presented a Newtonian behavior and that their viscosity, despite being higher for HPMC-based materials, was suitable for a proper spraying application. The surface tension of these fluids and the surface energies of the films resulting from them were assessed. Those values were similar among them and lower than the surface tension and surface energy of blood and dry skin, respectively, indicating their ability to spread and adhere to those surfaces. Considering the biological characterization, all the materials revealed a thrombogenic character, which suggests their positive impact on wound care by assisting in clotting and, therefore, reducing bleeding. Moreover, all materials were shown to be biocompatible when incubated with fibroblasts, achieving a minimum of 90% cellular viability.

Overall, this study allowed us to verify that the spray formulations developed meet the established criteria for the intended application and are promising tools for the treatment of injuries in wound first-aid care situations.

## 4. Materials and Methods

### 4.1. Materials

Hydroxypropyl methylcellulose (HPMC, Mn 10 kDa) and hydroxypropyl cellulose (HPC, Mn 100 kDa) were purchased from Alfa Aesar (Berlin, Germany). Ethanol (96%) was supplied by JMGS (Odivelas, Portugal). Anticoagulated rabbit blood (ACD blood), used in the thrombogenicity tests, was bought from PROBIOLÓGICA (Biologic Products Company; Lisbon, Portugal) and used on the same day it was received. Fetal bovine serum (FBS) was purchased from Biochrom AG (Berlin, Germany). Human Fibroblast Cells (Normal Human Dermal Fibroblasts adult) were acquired from PromoCell (Labclinics, S.A., Barcelona, Spain). Formamide (99%), ethylene glycol (99.8%), formaldehyde (99%), polyethylene glycol (PEG, Mn~400 Da), polyvinyl pyrrolidone (PVP, Mn~10 kDa), Dulbecco’s modified Eagle’s medium (DMEM-F12), ethylenediaminetetraacetic acid (EDTA), gentamicin, phosphate-buffered saline solution (PBS), and trypsin were acquired from Sigma-Aldrich (Sintra, Portugal). 3-(4,5-Dimethylthiazol-2-yl)-5-(3carboxymethoxyphenyl)-2-(4-sulphofenyl)-2H-tetrazolium, inner salt (MTS) was purchased from Promega (Madison, WI, USA).

### 4.2. Methods

#### 4.2.1. Polymeric Solutions and Films Preparation

Each polymer was dissolved in ethanol for 20 min and by continuous magnetic stirring at room temperature. Afterward, 4 different polymer proportion solutions were prepared, and a plasticizer (PEG, 1% *v*/*v*) was added to each formulation. Table 4 shows their composition.

Polymeric films were obtained by solvent evaporation (casting) from the prepared polymeric solutions. Each formulation was poured into a proper container and sprayed 5 times over glass microscopy slides, and fully formed films were obtained after 90 s. For DMTA characterization and surface energy determinations, 50 mL of each formulation solution was poured into glass Petri dishes and placed in an oven at 37 °C for 24 h to ensure the ethanol’s complete evaporation.

#### 4.2.2. Evaluation of Spraying Suitability and In Vitro Adhesion to Biological Substrates

Each polymer was dissolved in ethanol for 20 min. Spraying suitability and consequent film formation were assessed. In order to establish a visual evaluation of this parameter, 10 mL spray bottles were filled with each formulation, which was then sprayed (a total of 5 sprays) over glass microscope slides. Since the solutions were transparent, in order to better visualize the spraying effect and the formation of the films, dyes (green for HPC-HPMC (3:3) and HPC-HPMC (4:4) and blue for HPC-PVP and HPC-PVP (5:3)) were added to the mixtures. Properties such as ease of spraying and films’ detachability and flexibility were qualitatively assessed. Moreover, their ability to adhere to biological tissues was also determined before further characterization. The adhesion capacity of the films to biological tissues was evaluated in vitro by spraying the polymeric solutions on a biological substrate (pig muscle) surface at room temperature. For this purpose, the same procedure was applied and the same amount of spays were performed covering the tested areas. After drying and consequent film formation, the ability to remove the formed films by washing with saline was also qualitatively evaluated. During this step of the experimental procedure, gentle cleaning with a gauze swab was performed under a continuous instillation of saline solution.

### 4.3. Characterization Techniques

#### 4.3.1. Rheology

Rheology studies were performed to understand the flow behavior by determining the viscosity and the shear stress under the influence of the strain variation. Samples were analyzed in a Haake model RS1 rheometer using cone/plate geometries type Z34 DIN Ti. The test parameters were defined as temperature (T) at 37 °C, simulating the physiological temperature; the rate of deformation ($\dot{\gamma }$) from 0.1 to 200 rpm; and the time (t) defined for 360 s. Throughout these 360 s, the strain rate was increased from 1 to 200 rpm. Afterward, the deformation rate was maintained at 200 rpm for a further 240 s.

#### 4.3.2. Solubility Test in Saline Solution

The ability to solubilize in an aqueous solution is one of the most important properties of the prepared films to allow easy removal from a topical lesion without damaging the tissues. To evaluate the solubility of the films in saline solution (0.9% *w*/*w* NaCl), the samples were placed on a net support inside a glass beaker with 50 mL of saline solution. The mixture was stirred, and the time required for solubilization was registered.

#### 4.3.3. Dynamic–Mechanical Thermal Analysis

Dynamic–mechanical thermal analysis (DMTA) was used to determine the thermal properties of the different films. Thick specimens (15.20 × 7.45 × 1.10 mm^3^) were analyzed using a Triton Tritec 2000 in the Single Cantilever Bending mode, with a standard heating rate of 5 °C·min^−1^ (temperature range between −40 °C and 140 °C), in multifrequency conditions (1, 5 and 10 Hz) and with a displacement of 0.05 mm. The glass transition temperature (T_g_) was determined as the peak in tan δ by Equation (1).

(1) $$tan\delta =\frac{E^{\prime }}{E^{\prime \prime }},$$

where E′ and E″ represent the storage and loss modulus, respectively.

#### 4.3.4. Surface Tension and Surface Free Energy Assessment

The surface tension of the liquid formulations was determined by the Young–Laplace method using Dataphysics OCA 20 equipment at room temperature. The measurements were performed by analyzing the profile of the formed drops in contact with air over time and by applying the Laplace–Young equation.

Moreover, the surface energies of the films prepared with each formulation were assessed by contact angle measurement. Surface free energy values ($\gamma_{s}$) as well as their dispersive ($\gamma_{s}^{D}$) and polar ($\gamma_{s}^{P}$) components were obtained according to the Owens–Wendt–Rabel and Kaelbe method (OWRK) by static contact angle measurements with three liquids: water, formamide, and ethylene glycol. The contact angles were measured on Dataphysics OCA 20 equipment at room temperature, and all measurements were performed on the air-facing surface of the films using the sessile drop method. A minimum of eight measurements on different points were performed on each sample, and the mean static contact angle and standard error were determined.

#### 4.3.5. Characterization of the Biological Properties

##### Thrombogenicity Assessment

The evaluation of thrombus formation on film surfaces was carried out using an adaptation of the gravimetric method of Imai and Nose, which allows quantification of the mass of a thrombus formed on the material’s surface [39]. Anticoagulated rabbit blood (ACD-blood) was used for this purpose [40]. To each sample, prepared in triplicate, was added 250 μL of blood on the surface and also on an empty glass petri dish, which acted as a positive control (K^+^). The blood clotting test started with the addition of 25 μL of a 0.1 M calcium chloride solution (CaCl_2_) and then incubated for 4 min at 37 °C. Afterward, the coagulation process was stopped by the addition of 5 mL of distilled water. The clots formed were fixed with 1 mL of a 37% formaldehyde solution; their surface was gently dried, and, finally, they were weighed. The percentage of thrombogenicity was determined by Equation (2).

(2) $$\%Thrombogenicity=\frac{m_{test}-m_{negativecontrol}}{m_{positivecontrol}-m_{negativecontrol}}\times 100,$$

where $m_{test}$ is the average mass of the formed clot in each sample (g), $m_{negativecontrol}$ is the average mass of the formed clot in the negative control (g), and $m_{positivecontrol}$ is the average mass of formed clot in the positive control (g).

##### Evaluation of Materials Cytotoxicity

Cellular viability/cytotoxicity on the materials was evaluated using 3-(4,5-dimethylthiazol-2-yl)-5-(3-carboxymethoxyphenyl)-2-(4-sulfophenyl)-2H-tetrazolium (MTS) according to ISO 10993-5:2009 [41]. Briefly, samples (n = 5) were placed in 96-well plates and then sterilized by UV irradiation (254 nm, 7 mW/cm^2^) for 1 h. Since the material is intended for skin application, normal human dermal fibroblasts (NHDF) were used at a density of 10 × 10^3^ cells/well. These cells were seeded into the wells containing the samples and incubated at 37 °C and 5% CO_2_ in a humidified atmosphere for 1 day. As a positive control (K^+^), cells were incubated with EtOH (70%), and for the negative control (K^−^), cells were incubated with the culture medium [41]. The incubation medium was replaced daily. The optical density (OD) of each sample was measured using an absorbance microplate reader (Biorad x Mark Microplate Spectrophotometer) at 490 nm. Cell viability was determined according to Equation (3):

(3) $$Cell\;Viability\;\left(\%\right)=\frac{{OD}_{t}}{{OD}_{c}}\times 100\%,$$

where ${OD}_{t}$ is the optical density of the cells incubated with the samples, and ${OD}_{c}$ is the optical density of the non-treated control cells.

## Figures and Tables

**Table 1:** Qualitative evaluation of the assessed properties for each formulation and resultant film: Good (+); Very good (++); Excellent (+++).

Formulation		Properties Assessment				
		Ease of Spraying	Detachability	Flexibility	Adhesion to Biological Substrate	Washability with Saline
(a) HPC-HPMC (3:3)	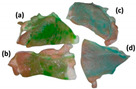	+++	+++	++	+++	++
(b) HPC-HPMC (4:4)		++	+++	++	+++	+
(c) HPC-PVP (4:4)		+++	++	+++	+++	+++
(d) HPC-PVP (5:3)		++	++	+++	+++	+++

**Table 2:** Solubilization time for each film.

Composition	Solubilization Time (min)
HPC-HPMC (3:3)	12
HPC-HPMC (4:4)	18
HPC-PVP (4:4)	4
HPC-PVP (5:3)	6

**Table 3:** Surface tension and surface energy values (with corresponding dispersive and polar components) for the developed polymeric films, dry skin, and blood.

Sample	Surface Tension (mN/m)	Surface Energy (mN/m)		
	$\gamma_{L}$	$\gamma_{s}$	$\gamma_{s}^{d}$	$\gamma_{s}^{d}$
Dry skin	-	43.7	35.7	8.0
Blood	47.5	-	-	-
HPC-HPMC (3:3)	17.21 ± 1.9	23.02 ± 2.2	5.04 ± 0.8	17.98 ± 2.1
HPC-HPMC (4:4)	22.45 ± 2.4	30.1 ± 2.6	1.34 ± 0.4	28.8 ± 2.5
HPC-PVP (4:4)	17.01 ± 0.8	21.8 ± 3.0	6.8 ± 1.4	15.0 ± 2.6
HPC-PVP (5:3)	19.89 ± 2.3	26.6 ± 2.0	2.6 ± 0.5	24.0 ± 1.9

**Table 4:** Formulations composition.

Formulation	HPC (%*w*/*w*)	HPMC (%*w*/*w*)	PVP (%*w*/*w*)	PEG (%*v*/*v*)
HPC-HPMC (3:3)	3	3	-	1
HPC-HPMC (4:4)	4	4	-	1
HPC-PVP (4:4)	4	-	4	1
HPC-PVP (5:3)	5	-	3	1

## Data Availability

The data presented in this study are available on request from the corresponding author.

## References

[1] Bhoyar S.D., Malhotra K., Madke B. (2023). Dressing Materials: A Comprehensive Review. J. Cutan. Aesthet. Surg..

[2] Atkin L., Stephenson J., Bateman S.D. (2015). Foam Dressings: A Review of the Literature and Evaluation of Fluid-Handling Capacity of Four Leading Foam Dressings. Wounds UK.

[3] Qu Z., Wang Y., Niu Q., Wen W., Ding G., Liu W. (2023). Alginate Dressings in Wound Care: A Systematic Review and Meta-Analysis of Randomized Clinical Trials. J. Clin. Exp. Dermatol. Res..

[4] Su L., Jia Y., Fu L., Guo K., Xie S. (2023). The Emerging Progress on Wound Dressings and Their Application in Clinic Wound Management. Heliyon.

[5] Liang Y., He J., Guo B. (2021). Functional Hydrogels as Wound Dressing to Enhance Wound Healing. ACS Nano.

[6] Gupta A., Kowalczuk M., Heaselgrave W., Britland S.T., Martin C., Radecka I. (2019). The Production and Application of Hydrogels for Wound Management: A Review. Eur. Polym. J..

[7] Rezvani Ghomi E., Khalili S., Nouri Khorasani S., Esmaeely Neisiany R., Ramakrishna S. (2019). Wound Dressings: Current Advances and Future Directions. J. Appl. Polym. Sci..

[8] Brumberg V., Astrelina T., Malivanova T., Samoilov A. (2021). Modern Wound Dressings: Hydrogel Dressings. Biomedicines.

[9] Daunton C., Kothari S., Smith L., Steele D. (2012). A History of Materials and Practices for Wound Management. Wound Pract. Res. Aust. J. Wound Manag..

[10] Bakhrushina E.O., Shumkova M.M., Sergienko F.S., Novozhilova E.V., Demina N.B. (2023). Spray Film-Forming Systems as Promising Topical in Situ Systems: A Review. Saudi Pharm. J..

[11] Brodovsky S., Dagan R., Ben-Bassatt M. (1986). Nobecutane Spray as a Temporary Dressing of Skin Graft Donor Sites. J. Dermatol. Surg. Oncol..

[12] Ellerker A.G. (1955). Nobecutane as a wound dressing. Lancet.

[13] Wu H., Du S., Lu Y., Li Y., Wang D. (2014). The Application of Biomedical Polymer Material Hydroxy Propyl Methyl Cellulose(HPMC) in Pharmaceutical Preparations. J. Chem. Pharm. Res..

[14] Takeuchi Y., Umemura K., Tahara K., Takeuchi H. (2018). Formulation Design of Hydroxypropyl Cellulose Films for Use as Orally Disintegrating Dosage Forms. J. Drug Deliv. Sci. Technol..

[15] Franco P., De Marco I. (2020). The Use of Poly(N-Vinyl Pyrrolidone) in the Delivery of Drugs: A Review. Polymers.

[16] Reddy K.S., Prabhakar M.N., Reddy V.N., Sathyamaiah G., Maruthi Y., Subha M.C.S., Chowdoji Rao K. (2012). Miscibility Studies of Hydroxypropyl Cellulose/Poly(Vinyl Pyrrolidone) in Dilute Solutions and Solid State. J. Appl. Polym. Sci..

[17] Koczkur K.M., Mourdikoudis S., Polavarapu L., Skrabalak S.E. (2015). Polyvinylpyrrolidone (PVP) in Nanoparticle Synthesis. Dalt. Trans..

[18] Yeh J., Chen C., Huang K.S., Nien Y.H., Chen J.L., Huang P.Z. (2006). Synthesis, Characterization, and Application of PVP/Chitosan Blended Polymers. J. Appl. Polym. Sci..

[19] Kadajji V.G., Betageri G.V. (2011). Water Soluble Polymers for Pharmaceutical Applications. Polymers.

[20] George H.F., Qureshi F. (2013). Newton’s Law of Viscosity, Newtonian and Non-Newtonian Fluids. Encyclopedia of Tribology.

[21] Sarode A., Wang P., Cote C., Worthen D.R. (2013). Low-Viscosity Hydroxypropylcellulose (HPC) Grades SL and SSL: Versatile Pharmaceutical Polymers for Dissolution Enhancement, Controlled Release, and Pharmaceutical Processing. AAPS PharmSciTech.

[22] Swei J., Talbot J.B. (2003). Viscosity Correlation for Aqueous Polyvinylpyrrolidone (PVP) Solutions. J. Appl. Polym. Sci..

[23] Osborne D.W., Mumper R.J. (2002). Atrix Laboratories Inc. Pharmaceutical Gel and Aerosol Formulations and Methods to Administer the Same to Skin and Mucosal Surfaces. US Patent.

[24] Kurakula M., Rao G.S.N.K. (2020). Pharmaceutical Assessment of Polyvinylpyrrolidone (PVP): As Excipient from Conventional to Controlled Delivery Systems with a Spotlight on COVID-19 Inhibition. J. Drug Deliv. Sci. Technol..

[25] Williams R.O., Sykora M.A., Mahaguna V. (2001). Method to Recover a Lipophilic Drug from Hydroxypropyl Methylcellulose Matrix Tablets. AAPS PharmSciTech.

[26] Mafi R., Mirabedini S.M., Attar M.M., Moradian S. (2005). Cure Characterization of Epoxy and Polyester Clear Powder Coatings Using Differential Scanning Calorimetry (DSC) and Dynamic Mechanical Thermal Analysis (DMTA). Prog. Org. Coat..

[27] Lim W.-S., Choi J.-W., Iwata Y., Koseki H. (2009). Thermal Characteristics of Hydroxypropyl Methyl Cellulose. J. Loss Prev. Process Ind..

[28] Buera M.D.P., Levi G., Karel M. (1992). Glass Transition in Poly(Vinylpyrrolidone): Effect of Molecular Weight and Diluents. Biotechnol. Prog..

[29] Turner D.T., Schwartz A. (1985). The Glass Transition Temperature of Poly(N-Vinyl Pyrrolidone) by Differential Scanning Calorimetry. Polymer.

[30] Mu Y., Sun Q., Wan X. (2023). Impact of Polymer Chemistry on the Application of Polyurethane/Ureas in Organic Thin Film Transistors. RSC Appl. Polym..

[31] Venkatraman S., Gale R. (1998). Skin Adhesives and Skin Adhesion. 1. Transdermal Drug Delivery Systems. Biomaterials.

[32] Agathopoulos S., Nikolopoulos P. (1995). Wettability and Interfacial Interactions in Bioceramic-body-liquid Systems. J. Biomed. Mater. Res..

[33] Eudier F., Savary G., Grisel M., Picard C. (2019). Skin Surface Physico-Chemistry: Characteristics, Methods of Measurement, Influencing Factors and Future Developments. Adv. Colloid Interface Sci..

[34] Braune S., Latour R.A., Reinthaler M., Landmesser U., Lendlein A., Jung F. (2019). In Vitro Thrombogenicity Testing of Biomaterials. Adv. Healthc. Mater..

[35] Zhao C., Liu X., Nomizu M., Nishi N. (2003). Blood Compatible Aspects of DNA-Modified Polysulfone Membrane—Protein Adsorption and Platelet Adhesion. Biomaterials.

[36] Cernadas T., Morgado S., Alves P., Gonçalves F.A.M.M., Correia T.R., Correia I.J., Ferreira P. (2020). Preparation of Functionalized Poly(Caprolactone Diol)/Castor Oils Blends to Be Applied as Photocrosslinkable Tissue Adhesives. J. Appl. Polym. Sci..

[37] Poussard L., Burel F., Couvercelle J.P., Lesouhaitier O., Merhi Y., Tabrizian M., Bunel C. (2005). In Vitro Thrombogenicity Investigation of New Water-Dispersible Polyurethane Anionomers Bearing Carboxylate Groups. J. Biomater. Sci. Polym. Ed..

[38] Santos J.M.C., Marques D.S., Alves P., Correia T.R., Correia I.J., Baptista C.M.S.G., Ferreira P. (2015). Synthesis, Functionalization and Characterization of UV-Curable Lactic Acid Based Oligomers to Be Used as Surgical Adhesives. React. Funct. Polym..

[39] Imai Y., Nose Y. (1972). A New Method for Evalution of Antithrombogenicity of Materials. J. Biomed. Mater. Res..

[40] (2017). Biological Evaluation of Medical Devices—Part 4: Selection of Tests for Interactions with Blood.

[41] (2009). Biological Evaluation of Medical Devices—Part 5: Tests for In Vitro Cytotoxicity.

